# Inhaled Therapy in Respiratory Disease: The Complex Interplay of Pulmonary Kinetic Processes

**DOI:** 10.1155/2018/2732017

**Published:** 2018-06-19

**Authors:** Jens Markus Borghardt, Charlotte Kloft, Ashish Sharma

**Affiliations:** ^1^Drug Discovery Sciences, Boehringer Ingelheim Pharma GmbH & Co. KG, Biberach an der Riss, Germany; ^2^Department of Clinical Pharmacy and Biochemistry, Institute of Pharmacy, Freie Universitaet Berlin, Berlin, Germany; ^3^Translational Medicine and Clinical Pharmacology, Boehringer Ingelheim Pharmaceuticals Inc., Ridgefield, CT, USA

## Abstract

The inhalation route is frequently used to administer drugs for the management of respiratory diseases such as asthma or chronic obstructive pulmonary disease. Compared with other routes of administration, inhalation offers a number of advantages in the treatment of these diseases. For example, via inhalation, a drug is directly delivered to the target organ, conferring high pulmonary drug concentrations and low systemic drug concentrations. Therefore, drug inhalation is typically associated with high pulmonary efficacy and minimal systemic side effects. The lung, as a target, represents an organ with a complex structure and multiple pulmonary-specific pharmacokinetic processes, including (1) drug particle/droplet deposition; (2) pulmonary drug dissolution; (3) mucociliary and macrophage clearance; (4) absorption to lung tissue; (5) pulmonary tissue retention and tissue metabolism; and (6) absorptive drug clearance to the systemic perfusion. In this review, we describe these pharmacokinetic processes and explain how they may be influenced by drug-, formulation- and device-, and patient-related factors. Furthermore, we highlight the complex interplay between these processes and describe, using the examples of inhaled albuterol, fluticasone propionate, budesonide, and olodaterol, how various sequential or parallel pulmonary processes should be considered in order to comprehend the pulmonary fate of inhaled drugs.

## 1. Introduction

Inhalation therapy has gained importance in recent decades [1]. Today, inhalation represents the administration route of choice for the delivery of drugs to treat respiratory disorders such as asthma and chronic obstructive pulmonary disease (COPD) [2–4]. The inhalation route is also being investigated in some instances for the systemic delivery of drugs [5, 6]. However, this review will focus on the inhalation route for local treatment of pulmonary diseases.

The primary goal of inhalation therapy for local treatment is to reduce pulmonary symptoms, for example, through the alleviation and/or prevention of airway inflammation and constriction [7]. Typical examples of inhaled drugs are corticosteroids, beta-sympathomimetics, muscarinic antagonists, and antibiotics. The inhalation of these drugs offers substantial benefits over their systemically administered formulations. Crucially, high pulmonary drug concentrations can be achieved by directly delivering the drug to the target organ, the lung. As a result, considerably lower inhaled doses can be therapeutically equivalent or even superior to higher doses of systemically administered therapy [8, 9]. High pulmonary yet low systemic drug concentrations conferred by inhalation lead to high pulmonary efficacy, while simultaneously reducing the risk of side effects. This means that higher systemic side effects are typically associated with orally or intravenously administered doses that would provide similar pulmonary efficacy compared with inhalation [7, 8, 10]. In addition to this favorable pulmonary efficacy to systemic safety ratio (“pharmacodynamic (PD) airway selectivity”), inhalation can provide a more rapid onset of effect in the lung than other routes of administration (within minutes for albuterol and up to half an hour for salmeterol) [11–13].

It is typically assumed that the concentration of a drug at its site of action determines efficacy. In the case of inhaled drugs for local treatment, this should mean that pulmonary concentrations drive efficacy. However, it is important to highlight that the lung should not be considered as a single, uniform organ; rather, it is an intricate organ with fine, branched architecture, and various specialized morphologic structures, including conducting airways, bronchioles, and alveoli [14, 15]. Due to the complexity of the lung, multiple pharmacokinetic (PK) processes exist that are specific to the pulmonary environment and the inhalation route, making pulmonary PK generally distinct and much more complex than those of drugs administered via other routes [16]. Drug concentrations in the lung are dependent on the interplay between various pulmonary kinetic processes, examples of which are outlined later in this review. All pulmonary processes are determined, or at least influenced, by one or more aspects of the inhaled medication, including physicochemical characteristics of the drug, drug formulation, and the inhalation device. Patient characteristics such as competency in inhalation technique may also impact on pulmonary efficacy [17]. Therefore, the design of inhaled drugs and development of inhalation products should be based on a sound understanding of the totality of all pulmonary PK processes. Along with pulmonary concentrations, systemic drug concentrations must be considered in order to evaluate and understand the systemic safety profile of an inhaled drug. However, systemic concentrations depend on a number of factors, including gastrointestinal absorption of the swallowed fraction of the drug, the rate and extent of absorption via pulmonary PK processes, and systemic disposition properties, such as distribution and elimination.

This review provides a detailed overview of the factors affecting drug inhalation and discusses both the specific pulmonary and systemic PK properties of inhaled drugs that ensure optimal performance. In addition, we will summarize important clinical examples (albuterol, fluticasone propionate/budesonide, and olodaterol) that clearly demonstrate the complex interplay between pulmonary PK processes and factors related to the drug, formulation and device, and patients.

## 2. Pulmonary Pharmacokinetic Processes following Inhalation in Contrast to Oral/Intravenous Administration

Traditional methods of administration, such as the oral and intravenous (IV) routes, have distinct PK considerations. Oral drugs must pass through the gastrointestinal tract, the portal vein, and the liver prior to absorption into the systemic circulation. Therefore, the rate and extent of absorption is initially influenced by the dissolution kinetics and the solubility in gastrointestinal fluid. After dissolution, the intestinal metabolism, passive permeability, and transporter affinity (efflux/active uptake) of the drug determine the uptake across the intestinal membrane. Last but not least, the transport in the portal vein, as well as metabolic and transporter capacity in hepatocytes, determines the systemic bioavailability of orally administered drugs (hepatic first pass) [18]. In contrast, IV administration involves injection or infusion of drug molecules directly into the blood vessels, thereby bypassing the gastrointestinal absorption step and the intestinal and hepatic first pass effects [19]. After either oral or IV administration, the target is often not located in the blood or plasma, and so the drug must distribute to the target organ, such as the lung in respiratory diseases. Therefore, after systemic administration, the plasma concentration-time profile is often considered as a surrogate concentration-time profile for the target organ and target site in the organ [20]. No drug transporters or relevant barriers between blood and the lung tissue are known, implying that free pulmonary concentrations are not higher than that in plasma; hence, pulmonary PK selectivity cannot be achieved by systemic administration.

**Figure 1:**
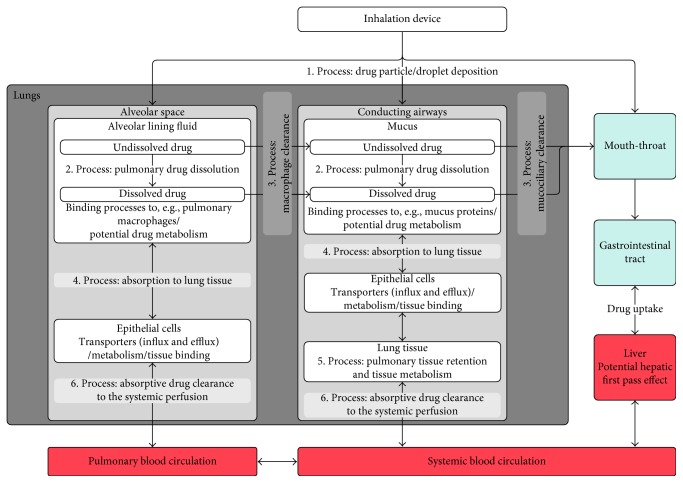
Summary of the lung-specific PK processes for inhaled drugs. Overview of the pulmonary-specific kinetic processes (1–6). The direction of the arrows indicates the direction of each process. For example, drug dissolution is considered to be a unidirectional process.

The pulmonary PK processes after drug inhalation contrast to those following systemic administration and comprise (1) drug particle or droplet deposition; (2) drug dissolution in the lung fluids; (3) mucociliary clearance in the conducting airways and macrophage clearance in the alveolar space; (4) absorption (of dissolved drug) to the lung tissue; (5) pulmonary tissue retention and potential pulmonary metabolism; and (6) absorptive drug clearance (drug transport) from the lung tissue to the systemic perfusion. The interplay between these processes is outlined in Figure 1 and will be further discussed later in the review. In addition to the complexity associated with the pulmonary PK processes, inhaled drugs present a challenge in that the quantification of pulmonary tissue concentrations by lung microdialysis, for example, is not feasible in a standard clinical setting. Given the multiple absorption processes following drug inhalation, plasma concentrations do not guarantee an easily interpretable surrogate concentration for pulmonary concentrations and PK processes. Rather, the plasma concentration of an inhaled drug is the result of drug absorption from different areas of the lung and gastrointestinal tract, as well as systemic drug disposition kinetics. Depending on a drug's physicochemical properties and first pass metabolism, absorption from the gastrointestinal tract can be particularly important when a significant fraction of inhaled drug gets swallowed. Overall, characterizing the PK of inhaled drugs is highly complex because both the pulmonary and systemic PK have to be simultaneously evaluated.

### 2.1. Step 1: Drug Particle/Droplet Deposition

**Figure 2:**
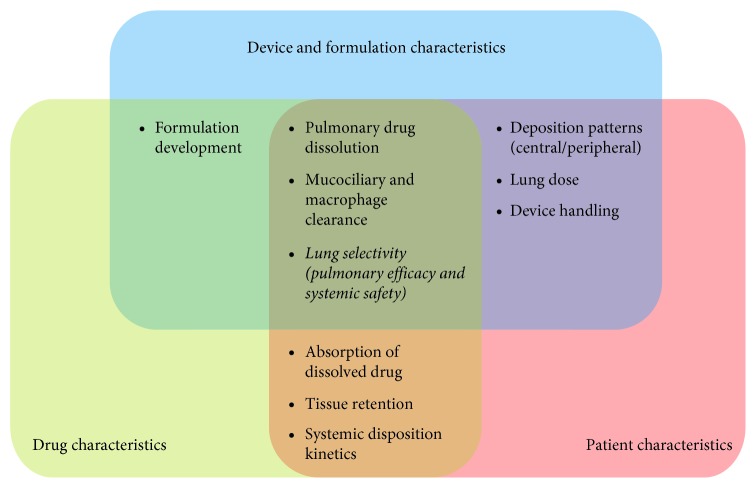
Schematic overview of the interplay of device and formulation, drug, and patient characteristics. The overlapping areas represent processes or parameters that are influenced or determined by the drug, the formulation/device, or the patient characteristics.

As summarized in Figure 1, the first process following inhalation is the deposition of drug particles or droplets. A fraction of the dose gets deposited in the device, whereas the remaining fraction of particles or droplets gets delivered to the respiratory system. As particles travel through the airway geometry, deposition occurs in the mouth-throat region, the conducting airways, and/or the alveolar space. The total fraction deposited in the lung is typically referred to as the “lung dose”, whereas the pulmonary deposition patterns are often described as being central (larger airways) or peripheral (smaller airways + alveolar space). Both the lung dose and the pulmonary deposition patterns are dependent on aerodynamic particle size, inhalation flow, device characteristics, and disease-related factors [10, 21–23] but are generally independent of the physicochemical characteristics of the drug itself [21] (Figure 2). Particles or droplets that fail to reach the lung and deposit in the mouth-throat region are subsequently swallowed [24, 25], whereas particles that reach the lung but fail to deposit are exhaled [10].

**Figure 3:**
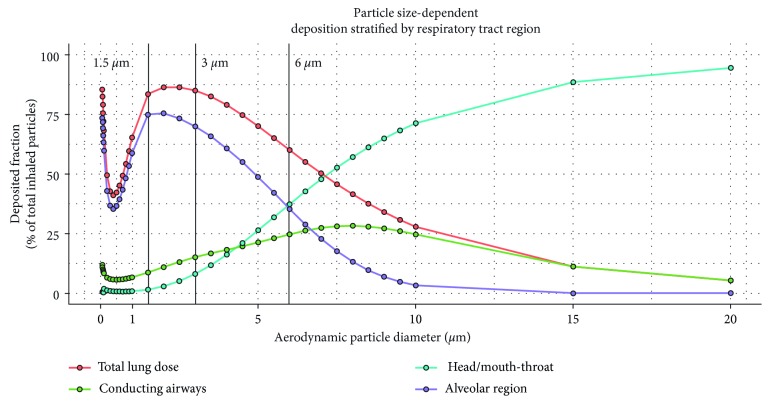
Particle size determines location of drug deposition. Aerodynamic particle size determines deposition patterns across the human respiratory tract. Simulations were performed using Multiple-Path Particle Dosimetry software [95]. Each simulated particle size represents one simulation. The Yeh/Schum five-lobe model [96] with uniform expansion was applied. The inhalation characteristics used for the simulation were an inhaled volume of 2 L, an inhalation flow rate of 60 L/min, and a breath-holding time of 8 seconds. These simulations do not account for the influence of an inhalation device on deposition.

One fundamental property that determines whether drug is deposited in the mouth-throat region or the airways is the aerodynamic particle diameter [21, 26]. Specifically, drug particles with an aerodynamic diameter of approximately 0.5–5 *µ*m have the greatest potential to be deposited in the lung (Figure 3). Smaller particles generally deposit more peripherally in the lung, such as in the alveolar space, whereas larger particles deposit more centrally, for example, in the large conducting airways. Particles larger than 5 *µ*m tend to deposit in the mouth-throat area [27], thereby reducing the lung dose. Deposition of larger particles in the central proximal airways and mouth-throat region is a result of inertial impaction, whereby maximum airflow velocity causes high mass particles to impact [28, 29]. Smaller particles are subject to sedimentation by gravity; this is the most effective mechanism of deposition in the small peripheral airways and can be enhanced by breath-hold following inhalation [21, 29]. Diffusional deposition, or Brownian motion, is most relevant to very small, submicron particles that deposit in the small airways and alveoli [21, 28].

Innovative inhalation devices have been designed to emit small particles (1–5 *µ*m aerodynamic diameter), thus maximizing the lung dose and the proportion of particles that are successfully delivered to their target site [30]. Inhalation flow and the velocity at which aerosol particles are emitted from a device and travel through the airways also have a strong impact on pulmonary deposition patterns. Generally, faster inhalation results in increased central and mouth-throat region deposition, whereas slower inhalation results in more peripheral deposition patterns. However, when using a low-resistance dry-powder inhaler, slow inhalation flow may be insufficient to disaggregate the powdered drug and can therefore limit lung deposition [31]. Overall, a design strategy for the inhalation device that couples slow-moving aerosol with smaller drug particles/droplets has so far been demonstrated as the most effective method in terms of total lung deposition and distal airway penetration [28, 32, 33].

### 2.2. Step 2: Pulmonary Drug Dissolution

Drug particles that have successfully deposited in the lung must dissolve into the fluids of the epithelial lining. As outlined in Figure 2, this process is dependent on drug formulation, physicochemical drug properties, and physiologic factors [25]. The conducting airways are lined with a biphasic gel-aqueous mucus layer, whereas alveoli are lined with alveolar lining fluid and pulmonary surfactant [34]. Both the thickness of the pulmonary lining fluid, which decreases from large to small airways [35], and its constitution can influence drug dissolution. While a mucus layer can act as a barrier to drug particles [36], surfactants produced by alveolar cells reduce surface tension and facilitate dissolution [37].

Pulmonary dissolution into the epithelial lining fluid, which is required for absorption, is also dependent on the properties of the inhaled drug [25]. While free, solubilized drugs may diffuse rapidly into the pulmonary lining fluid, dissolution characteristics are crucial for the absorption of drug deposited as particulate matter. Slow dissolution as a rate-limiting step can be desirable in that it prolongs lung retention and effect duration, albeit with a concomitant increase in the probability that drug particles get cleared by mucociliary clearance. For example, fluticasone, an inhaled corticosteroid, is a very hydrophobic drug characterized by slow dissolution kinetics that offers extended lung retention and long-lasting pulmonary efficacy [38–40]. In contrast, albuterol, a short-acting *β*_2_-agonist (SABA), is an example of a drug with high aqueous solubility and a faster pulmonary dissolution rate [41]. The desired dissolution profile therefore can influence the design of the drug formulation and the respective inhalation device.

### 2.3. Step 3: Mucociliary Clearance and Macrophage Clearance

The pulmonary bioavailability of deposited drug particles depends on several aspects, including the deposited lung dose and lung-specific clearance processes. In the conducting airways, the removal of drug particles is primarily achieved through mucociliary clearance, an evolutionary pulmonary protection mechanism against bacteria and dust particles. Here, the upward movement of mucus is driven by the beating of underlying cilia towards the pharynx. Drug particles transported to the pharynx via mucociliary clearance are subsequently swallowed and pass into the gastrointestinal tract [42]. Given that the velocity of mucociliary clearance increases with a wider airway diameter and thicker mucus layer, this process is fastest in the large airways [43]. Therefore, drug particles initially deposited in the central airways are cleared most quickly. Drug initially deposited in the peripheral conducting airways may be transported upwards and become available for absorption in the central airways. This process may confer long-term efficacy in the upper airways despite the rapid removal of drug particles from this region. In patients, the mucociliary clearance might be decreased due to increased thickness of the mucus layer or a higher mucus viscosity [44, 45]. In summary, the relevance of the mucociliary clearance is highly complex, being influenced by all the formulation, the drug characteristics, and the patient characteristics (Figure 2).

Particle clearance may also occur from the alveoli, where deposited particles can be phagocytosed by alveolar macrophages and cleared by transport to the lung-draining lymph nodes [46, 47]. Compared with mucociliary clearance, macrophage clearance of drug particles is far slower [48–50]. Therefore, macrophage clearance is typically assumed to be negligible for inhaled drugs, which dissolve before being cleared by this mechanism unless the drug is degraded by alveolar macrophages [25].

### 2.4. Step 4: Absorption to Lung Tissue

**Figure 4:**
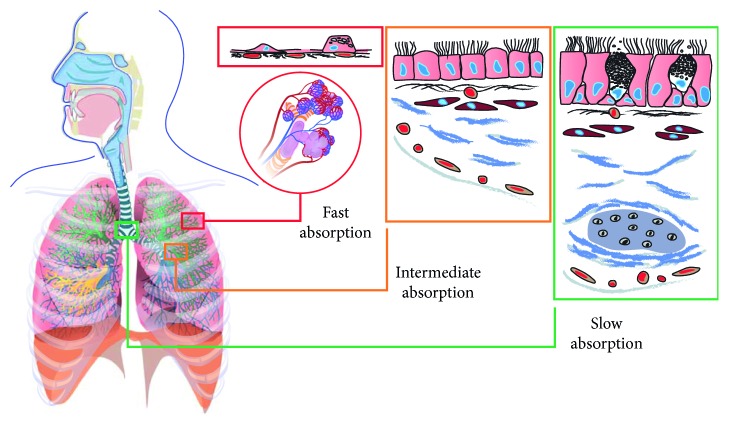
Summary of pulmonary absorption kinetics based on local physiologic characteristics of respiratory tract regions.

Drug particles that successfully evade pulmonary clearance mechanisms and dissolve in the epithelial lining fluid may then be absorbed into the lung tissue, a process that is proposed to occur by several mechanisms. The absorption through pulmonary barriers depends on both patient-specific airway characteristics and drug characteristics (Figure 2). Lipophilic drugs are thought to be rapidly absorbed after dissolution by passive transcellular diffusion through epithelial cells [51]. For small hydrophilic compounds, paracellular diffusion across the epithelium may occur through aqueous pores in intercellular gap junctions [51, 52]. Particles may also be absorbed through pores in the epithelium that arise transiently due to apoptosis [51]. Physiologic characteristics of the pulmonary environment may further influence pulmonary absorption (Figure 4). For instance, drug absorption takes place over an enormous surface area in the alveolar space but across a smaller surface in the conducting airways [10]. The very high perfusion of the alveolar space in comparison to the conducting airways also promotes a higher absorption rate in the alveolar space [10, 51]. Last but not least, an increased absorption rate in the alveolar space is conferred by the 0.2 *µ*m-thick epithelial cell monolayer, which is much thinner in comparison with the conducting airways [10, 53]. Overall, absorption of dissolved drug is considered fast in the alveoli and slow in the conducting airways due to differences in surface area, perfusion, and epithelial thickness [10, 53–56].

### 2.5. Step 5: Pulmonary Tissue Retention and Tissue Metabolism

Pulmonary tissue retention and distribution of absorbed/dissolved drug in the airways may be influenced by the physicochemical drug characteristics [57, 58] of inhaled drugs or patient-specific characteristics of airways (Figure 2). The tissue affinity, or pulmonary tissue partition coefficient, of an inhaled drug is likely the most important of these characteristics [25]. For example, basic drug molecules are reported to be retained in the lung by lysosomal trapping [58–60]. The low permeability of inhaled drugs such as long-acting muscarinic receptor antagonists (LAMAs) or long-acting *β*_2_-agonists (LABAs) is also considered to result in long lung retention [58, 60–62]. Further potential mechanisms that may increase pulmonary retention time include slow receptor off-kinetics [26, 63, 64], esterification in the lung tissue [65], and interaction with membrane lipid bilayers, the latter of which is proposed to account for the long duration of bronchodilation provided by the LABAs salmeterol and formoterol [66]. A long pulmonary retention time is desirable for most inhaled drugs to extend the duration of efficacy [26]. In addition to absorption from the pulmonary tissue into systemic circulation, drug absorbed in the lung can be redistributed to other regions of the airways from the systemic perfusion.

The lung also contains drug-metabolizing enzymes, although metabolic capacity for most enzymes in the lung is lower compared with the gastrointestinal and hepatic enzyme capacity [67, 68]. However, there are also enzymes that have a relevant capacity in the lung, such as CYP 1A1 in smokers [69, 70] or CYP 2E1 [71].

### 2.6. Step 6: Absorptive Drug Clearance to the Systemic Perfusion

The final pulmonary PK process is pulmonary clearance of drug from the lung tissues to the blood circulation (also known as absorptive clearance), a process that is heavily dependent on perfusion. The lung is the highest perfused organ in the body, as the complete cardiac output flows through the alveolar vascular bed [72]. Levels of local perfusion, however, vary between the different structures in the lung and are highest in the alveolar region. Here, the large absorption area, thin epithelium, and pulmonary circulation supply mean that absorptive clearance occurs more quickly compared with other regions of the lung [10, 73]. By contrast, perfusion is much slower in the conducting airways, which have a smaller surface area available for absorption and are supplied by the systemic circulation instead of the pulmonary circulation [72, 73]. In the alveoli, a high perfusion rate confers rapid equilibration with the systemic circulation and a very short half-life of drug distribution in this region. This was discussed for fluticasone propionate and salmeterol, resulting in reduced airway selectivity in the alveolar space compared with the conducting airways [73, 74]. Faster drug absorption as a general observation in the alveolar space was suggested by Brown and Schanker [75]. For this reason, and even though tissue retention in the alveolar space might not be completely absent, the tissue retention in the alveolar space was not included in Figure 1. In the tracheobronchial region, a lower perfusion rate combined with higher tissue retention offers a longer equilibration time and increased local airway selectivity [73, 74].

## 3. Systemic and Pulmonary Pharmacokinetic Properties of Inhaled Drugs

To minimize systemic exposure after drug inhalation, inhaled drugs should have low oral bioavailability and high systemic clearance [76, 77]. High oral bioavailability of an inhaled drug would result in efficient absorption of swallowed fractions and lead to higher systemic exposure. Therefore, to maximize airway selectivity, oral bioavailability should remain low, as this can affect the bioavailable fraction of swallowed drug and impact negatively on airway selectivity, thereby increasing the risk of systemic side effects. In addition, the systemic clearance of inhaled drugs should be high, as drugs with high systemic clearance have lower systemic exposure and are associated with high airway selectivity [26, 77]. These aspects further highlight the difference between inhaled drugs and orally administered drugs, as these are often characterized by a high oral bioavailability and a low systemic clearance.

It has also been discussed that high plasma protein binding might reduce initial high free plasma concentrations after pulmonary drug absorption, which are potentially associated with systemic adverse effects [78]. However, high plasma protein binding would likely correlate to lower free pulmonary concentrations, and so this hypothesis requires further evaluation.

Overall, inhaled drugs are most effective when they are designed for, and delivered to, their target location in the airways. An optimal inhaled drug combined with a well-designed inhalation device would confer the largest difference between pulmonary drug concentrations and systemic drug concentrations. This difference is known as (PK) airway selectivity, a concept that underpins the aim of respiratory treatment to specifically target the airways [77]. Ultimately, airway selectivity should also result in a high pulmonary PD selectivity. Airway selectivity can be further enhanced by optimizing particle size, which, as discussed, is a key determinant of the pulmonary regions in which inhaled drugs are deposited [21, 26–29, 79].

## 4. Well-Characterized Clinical PK and/or PD Examples

Drug concentrations in the lung typically cannot be directly measured. Therefore, to indirectly infer on the pulmonary concentration-time profiles, a combination of PK data after inhalation, oral and IV administration is required. Furthermore, a mathematical approach to simultaneously integrate all these data and to infer on the relevant pulmonary and systemic kinetic processes is required. Often, it can also be of importance to base these mathematic modelling approaches not only on *in vivo* PK data but additionally consider high-quality *in vitro* data, for example, about pulmonary drug deposition or pulmonary drug dissolution [80]. Even though this integration of data has provided valuable insights into the overall pulmonary PK profile for inhaled drugs, our understanding of the intricacies of the inhalation route remains more limited in comparison to traditional oral and IV routes [25].

Three PK and/or PD examples, namely, (1) albuterol, (2) fluticasone propionate/budesonide, and (3) olodaterol, will be discussed in detail below [81–85]. Further PK evaluations have also been published for inhaled drugs such as glycopyrronium [61], a LAMA, and AZD5423, a nonsteroidal glucocorticoid receptor modulator [86]. However, these are beyond the scope of this review.

### 4.1. Example 1: Pulmonary Efficacy of Inhaled Albuterol

As outlined before, particle size is a key determinant of overall drug deposition in the airways and therefore has a major impact on efficacy [21, 26]. Fine particles, that is, in the range of 1–5 *µ*m, are typically characterized by a low mouth-throat deposition and consequently by a high lung dose. Larger particles are deposited to a higher extent in the mouth-throat area, and subsequently a higher fraction of the inhaled drug is swallowed. In a study of monodisperse albuterol aerosols (1.5, 3.0, and 6.0 *µ*m) in patients with asthma, improved bronchodilation was achieved with larger particles that resulted in lower lung doses compared with an equivalent dose of smaller particles [87]. This was proposed to be a result of increased central deposition in the conducting airways, where smooth muscle, the target of *β*_2_-agonists, is prominent. Predicted deposition patterns of the three investigated particle sizes are highlighted in Figure 3. The overall smaller dose deposited in the lung for the 6 *µ*m sized particles also resulted in a lower systemic exposure. This example shows that, while a higher lung dose is typically considered to improve lung selectivity, a lower lung dose with an optimal deposition pattern resulted in higher efficacy and lower systemic exposure. In contrast, for idiopathic pulmonary fibrosis patients, a more peripheral deposition pattern was considered valuable, as potential targets are located more peripherally in the lung [88]. However, as outlined before, it remains to be demonstrated that the pulmonary selectivity in peripheral areas of the lung can be sufficient for future drug targets. In summary, the local pulmonary deposition patterns should be optimized with regard to the target location and the diseased area rather than just the lung dose or the fine particle fraction.

### 4.2. Example 2: Pulmonary PK and Efficacy of Inhaled Corticosteroids

**Figure 5:**
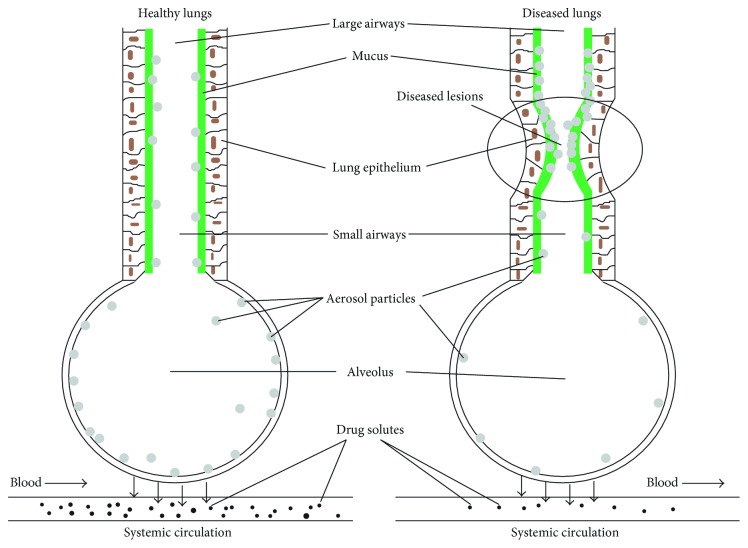
Inhaled drug particle deposition in healthy versus diseased lungs. Reproduced with permission from Wang et al. [23].

Disease-related factors have been shown to have strong effects on drug PK and PD behavior. In patients with obstructive pulmonary diseases (e.g. asthma or COPD), deposition is typically more central compared with less obstructed airways in healthy volunteers (Figure 5) [23, 89]. In multiple studies comparing the PK of fluticasone propionate and budesonide following inhalation by asthma or COPD patients with healthy volunteers, plasma concentrations of fluticasone propionate were lower in asthma and COPD patients compared with healthy volunteers, whereas those for budesonide were similar between patients and healthy volunteers [82–84]. A potential explanation is the combination of the slow dissolution of fluticasone propionate combined with a high central deposition in patients. This could lead to a higher fraction of the drug to be cleared by the mucociliary clearance in patients compared with healthy volunteers, and consequently, a lower fraction of deposited drug being absorbed in the lung. For bronchodilating drugs or quickly dissolving corticosteroids, as in this case budesonide, the difference in pulmonary PK and resulting systemic concentrations is less pronounced [82, 85]. These drugs dissolve faster in the lung fluids compared with fluticasone propionate and might, therefore, already be absorbed to the pulmonary tissue before a substantial amount of particles can be cleared by the mucociliary clearance. This would also result in a higher fraction of the initially deposited lung dose being available in a dissolved state at the target site. These findings suggest superior availability of budesonide compared with fluticasone propionate in acute asthma, in which the airways are markedly narrowed. This hypothesis is supported by the finding that inhaled fluticasone propionate confers a poor response in children with acute severe asthma [90]. On first sight, this might be surprising given that fluticasone propionate is considered to be an optimized drug for inhalation. Furthermore, the higher cleared fraction of fluticasone propionate in patients compared with healthy volunteers might be unexpected given that mucociliary clearance is typically slower in patients with airway diseases [91].

### 4.3. Example 3: Pulmonary PK of Inhaled Olodaterol

Olodaterol is another inhaled drug reported to have interesting PK characteristics in patients. The impact of asthma and COPD on the pulmonary PK characteristics of olodaterol was evaluated in a population PK analysis. Here, despite the decreased lung function in asthma and COPD patients, pulmonary bioavailable fractions of inhaled olodaterol were comparable with healthy volunteers [85]. Although this result is consistent with the established lung dose for the soft mist inhaler used [92], overall pulmonary absorption was slower in patients [85]. This is unexpected given that airway epithelia are typically damaged in asthma patients [93], and the integrity of tight junctions is compromised in COPD patients [94]. The overall slower absorption was discussed to be a result of the more central deposition in patients, which confers an extended pulmonary residence time. This suggests preferable lung targeting of inhaled olodaterol in asthma and COPD patients compared with healthy volunteers [85].

## 5. Conclusion

Inhaled drugs are the mainstay of treatment in the care of pulmonary diseases such as asthma and COPD [2–4]. Compared with other routes of administration, respiratory drugs that are specifically designed for inhalation can offer significant benefits, including direct delivery to the disease target site, rapid onset of action, high and long-term pulmonary efficacy, and reduced risk of systemic side effects [7]. These benefits can be achieved in drug design by considering the physiochemical properties of inhaled drugs (e.g., solubility), the device and formulation characteristics (e.g., particle size), and also the influence of patient characteristics. Overall, a sound understanding of the lung and its associated kinetic processes is necessary to overcome the complex challenges of the inhalational route of administration. Furthermore, the interplay between all pulmonary kinetic processes is highly complex. All pulmonary kinetic processes must be simultaneously considered as consideration of only a single process or parameter, such as lung dose, can lead to incorrect assumptions or inferences regarding pulmonary efficacy of inhaled drugs.

## References

[1] Crompton G. (2006). A brief history of inhaled asthma therapy over the last fifty years. *Primary Care Respiratory Journal*.

[2] Lavorini F., Mannini C., Chellini E. (2015). Challenges of inhaler use in the treatment of asthma and chronic obstructive pulmonary disease. *EMJ Respiratory*.

[3] Global Initiative for Asthma (2017). *GINA Report: Global Strategy for Asthma Management and Prevention*.

[4] Global Initiative for Chronic Obstructive Lung Disease (2017). *Global Strategy for the Diagnosis, Management, and Prevention of Chronic Obstructive Pulmonary Disease*.

[5] Henry R. R., Mudaliar S. R., Howland W. C. (2003). Inhaled insulin using the AERx insulin diabetes management system in healthy and asthmatic subjects. *Diabetes Care*.

[6] Sakagami M. (2004). Insulin disposition in the lung following oral inhalation in humans: a meta-analysis of its pharmacokinetics. *Clinical Pharmacokinectics*.

[7] Wright J., Brocklebank D., Ram F. (2002). Inhaler devices for the treatment of asthma and chronic obstructive airways disease (COPD). *Quality and Safety in Health Care*.

[8] Mash B., Bheekie A., Jones P. W. (2001). Inhaled vs oral steroids for adults with chronic asthma. *Cochrane Database of Systematic Reviews*.

[9] Walker S. R., Evans M. E., Richards A. J., Paterson J. W. (1972). The clinical pharmacology of oral and inhaled salbutamol. *Clinical Pharmacology and Therapeutics*.

[10] Labiris N. R., Dolovich M. B. (2003). Pulmonary drug delivery. part I: physiological factors affecting therapeutic effectiveness of aerosolized medications. *British Journal of Clinical Pharmacology*.

[11] Webb J., Rees J., Clark T. J. (1982). A comparison of the effects of different methods of administration of beta-2-sympathomimetics in patients with asthma. *British Journal of Diseases of the Chest*.

[12] van Noord J. A., Smeets J. J., Maesen F. P. (1998). A comparison of the onset of action of salbutamol and formoterol in reversing methacholine-induced bronchoconstriction. *Respiratory Medicine*.

[13] Palmqvist M., Persson G., Lazer L. (1997). Inhaled dry-powder formoterol and salmeterol in asthmatic patients: onset of action, duration of effect and potency. *European Respiratory Journal*.

[14] Itoh H., Nishino M., Hatabu H. (2004). Architecture of the lung: morphology and function. *Journal of Thoracic Imaging*.

[15] Herriges M., Morrisey E. E. (2014). Lung development: orchestrating the generation and regeneration of a complex organ. *Development*.

[16] Weber B., Hochhaus G. (2013). A pharmacokinetic simulation tool for inhaled corticosteroids. *AAPS Journal*.

[17] Ibrahim M., Verma R., Garcia-Contreras L. (2015). Inhalation drug delivery devices: technology update. *Medical Devices*.

[18] Zhao Y. H., Abraham M. H., Le J. (2002). Rate-limited steps of human oral absorption and QSAR studies. *Pharmaceutical Research*.

[19] Jin J. F., Zhu L. L., Chen M. (2015). The optimal choice of medication administration route regarding intravenous, intramuscular, and subcutaneous injection. *Patient Preference and Adherence*.

[20] Jones H. M., Rowland-Yeo K. (2013). Basic concepts in physiologically based pharmacokinetic modeling in drug discovery and development. *CPT: Pharmacometrics and Systems Pharmacology*.

[21] Tsuda A., Henry F. S., Butler J. P. (2013). Particle transport and deposition: basic physics of particle kinetics. *Comprehensive Physiology*.

[22] Thorsson L., Geller D. (2005). Factors guiding the choice of delivery device for inhaled corticosteroids in the long-term management of stable asthma and COPD: focus on budesonide. *Respiratory Medicine*.

[23] Wang Y. B., Watts A. B., Peters J. I., Williams R. O. (2014). The impact of pulmonary diseases on the fate of inhaled medicines–a review. *International Journal of Pharmaceutics*.

[24] Chrystyn H. (2001). Methods to identify drug deposition in the lungs following inhalation. *British Journal of Clinical Pharmacology*.

[25] Borghardt J. M., Weber B., Staab A., Kloft C. (2015). Pharmacometric models for characterizing the pharmacokinetics of orally inhaled drugs. *AAPS Journal*.

[26] Edsbacker S., Johansson C. J. (2006). Airway selectivity: an update of pharmacokinetic factors affecting local and systemic disposition of inhaled steroids. *Basic and Clinical Pharmacology and Toxicology*.

[27] Sheth P., Stein S. W., Myrdal P. B. (2015). Factors influencing aerodynamic particle size distribution of suspension pressurized metered dose inhalers. *AAPS PharmSciTech*.

[28] Bonini M., Usmani O. S. (2015). The importance of inhaler devices in the treatment of COPD. *COPD Research and Practice*.

[29] Thompson P. J. (1998). Drug delivery to the small airways. *American Journal of Respiratory and Critical Care Medicine*.

[30] Labiris N. R., Dolovich M. B. (2003). Pulmonary drug delivery. part II: the role of inhalant delivery devices and drug formulations in therapeutic effectiveness of aerosolized medications. *British Journal of Clinical Pharmacology*.

[31] Dal Negro R. W. (2015). Dry powder inhalers and the right things to remember: a concept review. *Multidisciplinary Respiratory Medicine*.

[32] Zierenberg B. (1999). Optimizing the in vitro performance of Respimat. *Journal of Aerosol Medicine*.

[33] Brand P., Hederer B., Austen G. (2008). Higher lung deposition with Respimat Soft Mist inhaler than HFA-MDI in COPD patients with poor technique. *International Journal of Chronic Obstructive Pulmonary Disease*.

[34] Ng A. W., Bidani A., Heming T. A. (2004). Innate host defense of the lung: effects of lung-lining fluid pH. *Lung*.

[35] Frohlich E., Mercuri A., Wu S., Salar-Behzadi S. (2016). Measurements of deposition, lung surface area and lung fluid for simulation of inhaled compounds. *Frontiers in Pharmacology*.

[36] Lai S. K., Wang Y. Y., Hanes J. (2009). Mucus-penetrating nanoparticles for drug and gene delivery to mucosal tissues. *Advanced Drug Delivery Reviews*.

[37] Akella A., Deshpande S. B. (2013). Pulmonary surfactants and their role in pathophysiology of lung disorders. *Indian Journal of Experimental Biology*.

[38] Daley-Yates P. T. (2015). Inhaled corticosteroids: potency, dose equivalence and therapeutic index. *British Journal of Clinical Pharmacology*.

[39] Johnson M. (1996). Pharmacodynamics and pharmacokinetics of inhaled glucocorticoids. *Journal of Allergy and Clinical Immunology*.

[40] Hogger P., Rawert L., Rohdewald P. (1993). Dissolution tissue binding and kinetics of receptor binding of inhaled glucocorticoidsn [abstract P1864]. *European Respiratory Journal*.

[41] Yalkowski S. H., He Y., Jain P. (2010). *Handbook of Aqueous Solubility Data*.

[42] Wanner A., Salathe M., O’Riordan T. G. (1996). Mucociliary clearance in the airways. *American Journal of Respiratory and Critical Care Medicine*.

[43] Foster W. M., Langenback E., Bergofsky E. H. (1980). Measurement of tracheal and bronchial mucus velocities in man: relation to lung clearance. *Journal of Applied Physiology: Respiratory, Environmental and Exercise Physiology*.

[44] Rogers F. G., Barnes P. J., Drazen J. M., Rennard S. I. (2009). Airway mucus hypersecretion in asthma and COPD: not the same?. *Asthma and COPD: Basic Mechanisms and Clinical Management*.

[45] Kim W. D. (1997). Lung mucus: a clinician’s view. *European Respiratory Journal*.

[46] Forbes B., O’Lone R., Allen P. P. (2014). Challenges for inhaled drug discovery and development: induced alveolar macrophage responses. *Advanced Drug Delivery Reviews*.

[47] Kirby A. C., Coles M. C., Kaye P. M. (2009). Alveolar macrophages transport pathogens to lung draining lymph nodes. *Journal of Immunology*.

[48] Camberlein E., Cohen J. M., Jose R. (2015). Importance of bacterial replication and alveolar macrophage-independent clearance mechanisms during early lung infection with *streptococcus pneumoniae*. *Infection and Immunity*.

[49] International Commission on Radiological Protection (1994). ICRP publication 66 human respiratory tract model for radiological protection. *Annals of the ICRP*.

[50] Cuddihy R. G., Fisher G. L., Kanapilly G. M. (1997). NCRP report no. 125-deposition, retention and dosimetry of inhaled radioactive substances. *Environment International*.

[51] Patton J. S. (1996). Mechanisms of macromolecule absorption by the lungs. *Advanced Drug Delivery Reviews*.

[52] Patton J. S., Fishburn C. S., Weers J. G. (2004). The lungs as a portal of entry for systemic drug delivery. *Proceedings of the American Thoracic Society*.

[53] Olsson B., Bondesson E., Borgstrom L., Smyth H., Hickey A. (2011). Pulmonary drug metabolism, clearance, and absorption. *Controlled Pulmonary Drug Delivery*.

[54] Patton J. S., Byron P. R. (2007). Inhaling medicines: delivering drugs to the body through the lungs. *Nature Reviews Drug Discovery*.

[55] Ruge C. A., Kirch J., Lehr C. M. (2013). Pulmonary drug delivery: from generating aerosols to overcoming biological barriers-therapeutic possibilities and technological challenges. *Lancet Respiratory Medicine*.

[56] Schanker L. S., Mitchell E. W., Brown R. A. (1986). Species comparison of drug absorption from the lung after aerosol inhalation or intratracheal injection. *Drug Metabolism and Disposition: The Biological Fate of Chemicals*.

[57] Backstrom E., Lundqvist A., Boger E. (2016). Development of a novel lung slice methodology for profiling of inhaled compounds. *Journal of Pharmaceutical Sciences*.

[58] Bäckström E., Boger E., Lundqvist A. (2016). Lung retention by lysosomal trapping of inhaled drugs can be predicted in vitro with lung slices. *Journal of Pharmaceutical Sciences*.

[59] MacIntyre A. C., Cutler D. J. (1988). The potential role of lysosomes in tissue distribution of weak bases. *Biopharmaceutics and Drug Disposition*.

[60] Borghardt J. M., Weber B., Staab A. (2016). Investigating pulmonary and systemic pharmacokinetics of inhaled olodaterol in healthy volunteers using a population pharmacokinetic approach. *British Journal of Clinical Pharmacology*.

[61] Bartels C., Looby M., Sechaud R., Kaiser G. (2013). Determination of the pharmacokinetics of glycopyrronium in the lung using a population pharmacokinetic modelling approach. *British Journal of Clinical Pharmacology*.

[62] Weber B., Troconiz I. F., Borghardt A., Staab A., Sharma A., Dalby R. N., Byron P. R., Young P. M. (2015). Model-based evaluation of single and multiple dose pharmacokinetics of inhaled tiotropium in healthy volunteers and implications for systemic exposure studies. *Respiratory Drug Delivery (RDD) Europe 2015*.

[63] Vauquelin G., Charlton S. J. (2010). Long-lasting target binding and rebinding as mechanisms to prolong in vivo drug action. *British Journal of Pharmacology*.

[64] Casarosa P., Kollak I., Kiechle T. (2011). Functional and biochemical rationales for the 24-hour-long duration of action of olodaterol. *Journal of Pharmacology and Experimental Therapeutics*.

[65] Jendbro M., Johansson C. J., Strandberg P. (2001). Pharmacokinetics of budesonide and its major ester metabolite after inhalation and intravenous administration of budesonide in the rat. *Drug Metabolism and Disposition*.

[66] Anderson G. P., Linden A., Rabe K. F. (1994). Why are long-acting beta-adrenoceptor agonists long-acting?. *European Respiratory Journal*.

[67] Anttila S., Hukkanen J., Hakkola J. (1997). Expression and localization of CYP3A4 and CYP3A5 in human lung. *American Journal of Respiratory Cell and Molecular Biology*.

[68] Hukkanen J., Pelkonen O., Hakkola J., Raunio H. (2002). Expression and regulation of xenobiotic-metabolizing cytochrome P450 (CYP) enzymes in human lung. *Critical Reviews in Toxicology*.

[69] Anttila S., Hietanen E., Vainio H. (1991). Smoking and peripheral type of cancer are related to high levels of pulmonary cytochrome P450IA in lung cancer patients. *International Journal of Cancer*.

[70] Omiecinski C. J., Redlich C. A., Costa P. (1990). Induction and developmental expression of cytochrome P450IA1 messenger RNA in rat and human tissues: detection by the polymerase chain reaction. *Cancer Research*.

[71] Shimada T., Yamazaki H., Mimura M. (1996). Characterization of microsomal cytochrome P450 enzymes involved in the oxidation of xenobiotic chemicals in human fetal liver and adult lungs. *Drug Metabolism and Disposition: The Biological Fate of Chemicals*.

[72] Brown R. P., Delp M. D., Lindstedt S. L. (1997). Physiological parameter values for physiologically based pharmacokinetic models. *Toxicology and Industrial Health*.

[73] Boger E., Evans N., Chappell M. (2016). Systems pharmacology approach for prediction of pulmonary and systemic pharmacokinetics and receptor occupancy of inhaled drugs. *CPT: Pharmacometrics and Systems Pharmacology*.

[74] Backstrom E., Hamm G., Nilsson A. (2018). Uncovering the regional localization of inhaled salmeterol retention in the lung. *Drug Delivery*.

[75] Brown R. A., Schanker L. S. (1983). Absorption of aerosolized drugs from the rat lung. *Drug Metabolism and Disposition: The Biological Fate of Chemicals*.

[76] Tayab Z. R., Hochhaus G. (2005). Pharmacokinetic/pharmacodynamic evaluation of inhalation drugs: application to targeted pulmonary delivery systems. *Expert Opinion on Drug Delivery*.

[77] Hochhaus G., Mollmann H., Derendorf H., Gonzalez-Rothi R. J. (1997). Pharmacokinetic/pharmacodynamic aspects of aerosol therapy using glucocorticoids as a model. *Journal of Clinical Pharmacology*.

[78] Colice G. L. (2006). Pharmacodynamic and pharmacokinetic considerations in choosing an inhaled corticosteroid. *Treatments in Respiratory Medicine*.

[79] Scichilone N., Benfante A., Bocchino M. (2015). Which factors affect the choice of the inhaler in chronic obstructive respiratory diseases?. *Pulmonary Pharmacology and Therapeutics*.

[80] Backman P., Arora S., Couet W. (2018). Advances in experimental and mechanistic computational models to understand pulmonary exposure to inhaled drugs. *European Journal of Pharmaceutical Sciences*.

[81] Usmani O. S., Biddiscombe M. F., Nightingale J. A. (2003). Effects of bronchodilator particle size in asthmatic patients using monodisperse aerosols. *Journal of Applied Physiology*.

[82] Harrison T. W., Wisniewski A., Honour J., Tattersfield A. E. (2001). Comparison of the systemic effects of fluticasone propionate and budesonide given by dry powder inhaler in healthy and asthmatic subjects. *Thorax*.

[83] Harrison T. W., Tattersfield A. E. (2003). Plasma concentrations of fluticasone propionate and budesonide following inhalation from dry powder inhalers by healthy and asthmatic subjects. *Thorax*.

[84] Singh S. D., Whale C., Houghton N. (2003). Pharmacokinetic and systemic effects of inhaled fluticasone propionate in chronic obstructive pulmonary disease. *British Journal of Clinical Pharmacology*.

[85] Borghardt J. M., Weber B., Staab A. (2016). Model-based evaluation of pulmonary pharmacokinetics in asthmatic and COPD patients after oral olodaterol inhalation. *British Journal of Clinical Pharmacology*.

[86] Melin J., Prothon S., Kloft C. (2017). Pharmacokinetics of the inhaled selective glucocorticoid receptor modulator AZD5423 following inhalation using different devices. *AAPS Journal*.

[87] Usmani O. S., Biddiscombe M. F., Barnes P. J. (2005). Regional lung deposition and bronchodilator response as a function of beta2-agonist particle size. *American Journal of Respiratory and Critical Care Medicine*.

[88] Usmani O. S., Biddiscombe M. F., Yang S. (2018). The topical study of inhaled drug (salbutamol) delivery in idiopathic pulmonary fibrosis. *Respiratory Research*.

[89] Darquenne C. (2012). Aerosol deposition in health and disease. *Journal of Aerosol Medicine and Pulmonary Drug Delivery*.

[90] Schuh S., Reisman J., Alshehri M. (2000). A comparison of inhaled fluticasone and oral prednisone for children with severe acute asthma. *New England Journal of Medicine*.

[91] Houtmeyers E., Gosselink R., Gayan-Ramirez G., Decramer M. (1999). Regulation of mucociliary clearance in health and disease. *European Respiratory Journal*.

[92] Brand P., Meyer T., Weuthen T. (2007). Lung deposition of radiolabeled tiotropium in healthy subjects and patients with chronic obstructive pulmonary disease. *Journal of Clinical Pharmacology*.

[93] Aoshiba K., Nagai A. (2004). Differences in airway remodeling between asthma and chronic obstructive pulmonary disease. *Clinical Reviews in Allergy and Immunology*.

[94] Shaykhiev R., Otaki F., Bonsu P. (2011). Cigarette smoking reprograms apical junctional complex molecular architecture in the human airway epithelium in vivo. *Cellular and Molecular Life Sciences*.

[95] Applied Research Associates (2009). *Multiple-Path Dosimetry Model*.

[96] Yeh H. C., Schum G. M. (1980). Models of human lung airways and their application to inhaled particle deposition. *Bulletin of Mathematical Biology*.

